# Enrichment of extracellular vesicles from human synovial fluid using size exclusion chromatography

**DOI:** 10.1080/20013078.2018.1490145

**Published:** 2018-06-26

**Authors:** Andrew D. Foers, Simon Chatfield, Laura F. Dagley, Benjamin J. Scicluna, Andrew I. Webb, Lesley Cheng, Andrew F. Hill, Ian P. Wicks, Ken C. Pang

**Affiliations:** a Inflammation Division, The Walter and Eliza Hall Institute of Medical Research, Parkville, Victoria, Australia; b Department of Medical Biology, University of Melbourne, Parkville, Victoria, Australia; c Department of Rheumatology, Royal Melbourne Hospital, Parkville, Victoria, Australia; d Department of Biochemistry and Genetics, La Trobe Institute for Molecular Science, La Trobe University, Bundoora, Victoria, Australia; e Department of Paediatrics, University of Melbourne, Parkville, Victoria, Australia; f Department of Psychiatry, University of Melbourne, Parkville, Victoria, Australia; g Genetics Theme, Murdoch Children’s Research Institute, Parkville, Victoria, Australia; h Department of Adolescent Medicine, Royal Children’s Hospital, Parkville, Victoria, Australia

**Keywords:** Synovial fluid, extracellular vesicles, ultracentrifugation, sucrose density gradient ultracentrifugation, size exclusion chromatography, high-density lipoprotein, serum albumin, fibronectin, extracellular matrix, rheumatoid arthritis

## Abstract

As a complex biological fluid, human synovial fluid (SF) presents challenges for extracellular vesicle (EV) enrichment using standard methods. In this study of human SF, a size exclusion chromatography (SEC)-based method of EV enrichment is shown to deplete contaminants that remain after standard ultracentrifugation-based enrichment methods. Specifically, considerable levels of serum albumin, the high-density lipoprotein marker, apolipoprotein A-I, fibronectin and other extracellular proteins and debris are present in EVs prepared by differential ultracentrifugation. While the addition of a sucrose density gradient purification step improved purification quality, some contamination remained. In contrast, using a SEC-based approach, SF EVs were efficiently separated from serum albumin, apolipoprotein A-I and additional contaminating proteins that co-purified with high-speed centrifugation. Finally, using high-resolution mass spectrometry analysis, we found that residual contaminants which remain after SEC, such as fibronectin and other extracellular proteins, can be successfully depleted by proteinase K. Taken together, our results highlight the limitations of ultracentrifugation-based methods of EV isolation from complex biological fluids and suggest that SEC can be used to obtain higher purity EV samples. In this way, SEC-based methods are likely to be useful for identifying EV-enriched components and improving understanding of EV function in disease.

## Introduction

Synovial fluid (SF) lubricates synovial joints to provide a low-friction environment that allows joint movement, and it also nourishes avascular articular cartilage. In diseases such as rheumatoid arthritis (RA), increased levels of extracellular vesicles (EVs) in SF have been observed [1]. Specific cells release large numbers of EVs in RA. For example, elevated levels of CD3+ and CD8+ T cell-derived and platelet-derived EVs are detected in RA SF [2,3]. Furthermore, SF EVs are described as having potent pro-inflammatory properties that might contribute to the perpetuation of joint inflammation in RA [1,3–5]. Understanding the composition and function of SF EVs is therefore of great interest and may provide insight into pathogenesis of joint diseases.

The composition and viscosity of SF present challenges for EV isolation. SF is an ultrafiltrate of plasma, combined with molecules secreted by synoviocytes and chondrocytes, including hyaluronan and proteoglycan 4 (lubricin), both of which increase SF viscosity. Studies on EVs isolated from SF have mainly used differential ultracentrifugation, where EVs are isolated using ultracentrifugation, with or without additional purification over a sucrose density gradient. These EV preparations display considerable levels of non-EV material, including free immunoglobulins, immune complexes and extracellular debris [2,6–9]. Similarly, ultracentrifuge-based EV enrichments from other complex biological fluids such as plasma also show high levels of contaminants, including high-density lipoproteins (HDLs) and serum albumin [10,11].

Size exclusion chromatography (SEC) (also known as gel filtration) has recently been described as an efficient means of EV purification from complex biological fluids, including blood/plasma [11,12], urine [13] and breast milk [14]. In SEC, EVs are separated from other material according to differences in hydrodynamic radii [15]. SEC is proposed to have advantages over ultracentrifugation-based methods as it avoids high centrifugal forces that are associated with vesicle rupture and aggregation [16,17]. SEC has also been shown to be capable of separating EVs from serum albumin [11].

Using human SF obtained from patients with inflammatory arthritis, we compared small EV enrichment (including exosomes) using differential ultracentrifugation (with and without further sucrose gradient purification) and SEC. Our results show that EV enrichments from SF using differential ultracentrifugation and sucrose density gradient ultracentrifugation co-purify serum albumin and HDLs. In contrast, EV enrichment by SEC is able to separate EVs from the albumin and HDLs present in SF. Extracellular matrix and immunological components that remained in a SEC EV enrichment from RA SF were further depleted with proteinase K treatment. This method should prove useful for examining the contents of SF EVs, and identifying pathogenic SF EV components.

## Methods

To assist interpretation and reproducibility, details of experimental procedures are available via EV-TRACK (ID: RJ0786OI) [18].

### Collection and storage of human synovial fluid

SF was obtained from RA patients undergoing arthro-centesis as part of routine clinical care, used with informed consent and the approval of the Melbourne Health Research and Ethics Committee (Projects 2005.056 and 2010.293). Following needle aspiration, SF was centrifuged at 2,000 x *g* for 20 min to remove cells, then aliquoted and stored at −80°C until the time of experiment.

### Sample preparation

**Figure 1.**
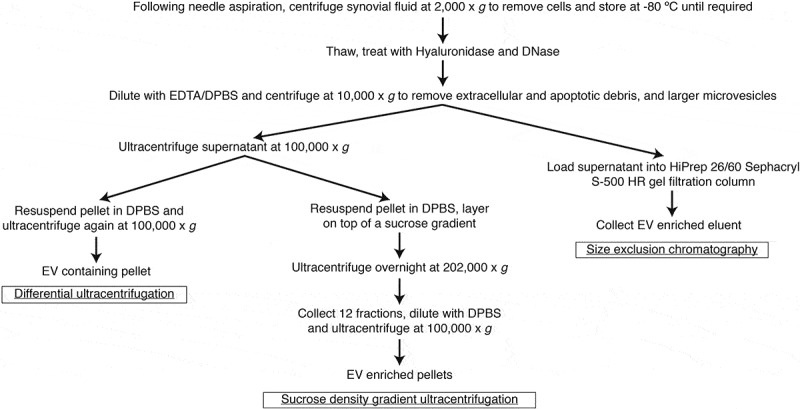
Workflow of SF sample preparation and EV enrichments by differential ultracentrifugation, sucrose density gradient ultracentrifugation and SEC.

To remove contaminating hyaluronan and DNA, cell-depleted SF was thawed and treated with Hyaluronidase (Sigma) at 30 U/ml (as described [8]), and DNase I (Worthington) at 20 U/ml for 15 min at 37°C prior to EV isolations. For differential ultracentrifugation and sucrose density gradient ultracentrifugation, 5 ml of enzyme treated, cell-depleted SF was diluted 1:4 with 4.84 mM EDTA/DPBS. For SEC, 5 ml of enzyme treated, cell-depleted SF was diluted to 13 ml with 4.84 mM EDTA/DPBS. Diluted samples were centrifuged at 10,000 x *g* (avg) (11,700 RPM, *k*-Factor = 1563) in a 70 Ti rotor using polycarbonate tubes (Beckman Coulter) for 30 min at 4°C to deplete extracellular and apoptotic debris and larger microvesicles. The supernatant was collected for EV enrichment by either differential ultracentrifugation, sucrose density gradient ultracentrifugation or SEC, as illustrated in Figure 1.

### EV enrichment by differential ultracentrifugation and sucrose density gradient ultracentrifugation

10,000 x *g* supernatant was transferred to fresh polycarbonate tubes and ultracentrifuged at 100,000 x *g* (avg) (36,900 RPM, *k*-Factor = 157) in a 70 Ti rotor for 90 min at 4°C to obtain a crude EV pellet. For EV enrichment by ultracentrifugation, the crude EV pellet was resuspended in 25 ml of DPBS and ultracentrifuged again at 100,000 x *g* (avg) (36,900 RPM, *k*-Factor = 157) in a 70 Ti rotor for 90 min at 4°C and the EV enriched pellet was collected. For EV enrichment by sucrose density gradient ultracentrifugation, the crude EV pellet was resuspended in 650 μl of 4.84 mM EDTA/DPBS and overlaid on a discontinuous sucrose gradient [2.0–0.25 M sucrose, 20 mM HEPES (pH 7.4)] and ultracentrifuged overnight at 202,000 x *g* (avg) (40,000 RPM, *k*-Factor = 137) in a SW 40 Ti rotor at 4°C in a thin wall Ultra-Clear^TM^ tube (Beckman Coulter). Twelve 1.05 ml aliquots were then collected from the top down and 50 μl of each fraction was taken to measure density on a Refracto 30GS refractometer (Mettler-Toledo). The remaining 1 ml aliquots were transferred to individual polycarbonate tubes, diluted 10-fold with DPBS and ultracentrifuged at 100,000 x *g* (avg) (38,200 RPM, *k*-Factor = 122) in a 70.1 Ti rotor for 90 min at 4°C. Pellets were resuspended in equal volumes of lysis buffer (150 mM NaCl, 50 mM Tris at pH 7.4, 1% (v/v) Triton X-100, 0.5% (w/v) sodium deoxycholate), containing a protease inhibitor cocktail (Roche) for western blot analysis, or with equal volumes of 4.84 mM EDTA/DPBS for transmission electron microscopy (TEM).

### EV enrichment by size exclusion chromatography

10,000 x *g* supernatant was loaded into a HiPrep 26/60 Sephacryl S-500 HR prepacked gel filtration column (GE Healthcare Life Sciences), which contains a hydrophilic, rigid allyl dextran/bisacrylamide matrix with a bed height/volume of 600 mm/120 ml, and eluted with 4.84 mM EDTA/DPBS at a flow rate of 1.5 ml/min. For TEM and nanoparticle tracking analysis (see the following text), EV-containing SEC fractions were assessed without concentration, unless specified otherwise. Where indicated, SEC fractions were concentrated by ultracentrifugation at 100,000 x *g* (avg) (36,900 RPM, *k*-Factor = 157) in a 70 Ti rotor for 90 min at 4°C, and pellets were resuspended either in lysis buffer containing a protease inhibitor cocktail for gel electrophoresis and western blot analysis, or in 4.84 mM EDTA/DPBS for TEM (see below). Alternatively, for some experiments, SEC fractions were concentrated via ultrafiltration by passing SEC fractions through an Amicon Ultra-15 100 kDa cellulose ultrafiltration device (Merck Millipore), according to the manufacturer’s instructions. Protein content was measured by Pierce BCA Protein Assay (ThermoFisher).

### Gel electrophoresis and western blot analysis

EV enrichments were lysed on ice for at least 10 min followed by addition of 4×SDS loading buffer (125 mM Tris-HCl at pH 6.8, 4% SDS (w/v), 20% glycerol (w/v), 0.02% bromophenol blue (w/v), with 8% β-mercaptoethanol). Samples were boiled at 95°C for 10 min and electrophoresed on precast NuPAGE 4–12% Bis-Tris protein gels (Invitrogen) at 150 V for 70 min. For Coomassie staining, gels were incubated with SimplyBlue SafeStain (ThermoFisher) using a microwave procedure, in accordance with the manufacturer’s protocol. For western blot analysis, gels were transferred onto 0.45 μm polyvinylidene difluoride membranes. About 3–5% skim milk was used for blocking and membranes were probed over-night at 4°C in 1% BSA/PBS-T (PBS containing 0.1% Tween 20) containing the relevant primary antibodies (Supplementary Table 1). Membranes were washed three times for 15 min in PBS-T and incubated at room temperature (RT) for 2 h with horseradish peroxidase (HRP)-conjugated secondary antibodies. Probed membranes were washed three times in PBS-T and after incubation with Immobilon Western Chemiluminescent HRP Substrate (Merck), imaged with a ChemiDoc Touch Imaging System (Bio-Rad Laboratories).

### Transmission electron microscopy

TEM was performed as previously described [19]. Briefly, purified EVs in 4.84 mM EDTA/DPBS were fixed with 1% glutaraldehyde overnight at 4°C and adsorbed onto glow-discharged 200 mesh formvar with carbon coating Cu grids (ProSciTech). Grids were washed twice with MilliQ water, negatively stained with 2% uranyl acetate and imaged on a Tecnai G2 F30 transmission electron microscope (FEI), operating at 300 kV.

### Nanoparticle tracking analysis (NanoSight)

SEC fractions were collected and stored overnight at 4°C prior to NanoSight analysis. Particle size and concentration were assessed on a NanoSight NS300 (Malvern Instruments). Each sample was analysed with the camera level optimised using the “Auto Setup” feature, and a detection threshold of 5. Five replicate videos of 30 s duration per fraction were collected and results averaged. Analysis was performed with NTA 3.2 Dev Build 3.2.16.

### Proteinase k treatment and trypsin digestion

SEC fractions 2 and 3 were pooled and concentrated by ultracentrifugation as described above. Pellets were resuspended in DPBS and incubated at 37°C with proteinase K (Roche) at 75 U/ml (based on haemoglobin assay) or an equivalent volume of diluent (H_2_O). Phenylmethylsulfonyl fluoride was added to both the proteinase K and the untreated samples at 0.625 mM and both samples were ultracentrifuged at 58,100* x g* (avg) (35,900 RPM, *k*-Factor = 157) for 90 min at 4°C in a polypropylene tube, using a TLA45 rotor. The EV pellet was then resuspended in lysis buffer. EVs were prepared for mass spectrometry analysis as previously described [20], but with several differences. Sera-Mag magnetic carboxylate modified beads (ThermoFisher) were prepared by rinsing with water three times prior to use and stored at 4**°**C at a stock concentration of 10 μg/μl. EV lysates were reduced with dithiothreitol at 50 mM for 1 h at 37**°**C. Samples were then alkylated with iodoacetamide at 100 mM for 30 min in the dark at RT. Samples were quenched with dithiothreitol at 250 mM, prior to the addition of 12 μl carboxylate bead stock, and acetonitrile to a final concentration of 70% (v/v). The beads were left to precipitate for 20 min at RT and then washed twice with 70% ethanol and once with acetonitrile. Beads were transferred to a 96 well plate and acetonitrile was completely evaporated from the sample prior to the addition of 40 μl digestion buffer (10% 2-2-2-trifluorethanol, 100 mM NH_4_HCO_3_) containing 1 μg Trypsin-gold (Promega) and 1 μg Lys-C (Wako) at a 1:25 enzyme:substrate ratio. The plate was briefly sonicated in a water bath to disperse the beads, and then transferred to a ThermoMixer instrument for digestion at 37**°**C for 1 h (1,200 RPM). The supernatant was then collected from the beads using a magnetic rack and an additional elution with 20 μl of 2% dimethyl sulfoxide (Sigma) was performed on the beads. Peptides were desalted on in-house made C18 stage tips (3M) as previously described [21], and lyophilised to dryness using a CentriVap (Labconco), prior to reconstitution in Buffer A (0.1% formic acid, 2% acetonitrile) for mass spectrometry analysis.

### Mass spectrometry (MS) analysis

Peptides were separated by reverse-phase chromatography on a 1.9 μm C18 fused silica column (I.D. 75 μm, O.D. 360 μm x 25 cm length) packed into an emitter tip (Ion Opticks), using a nano-flow HPLC (M-class, Waters). The HPLC was coupled to an Impact II UHR-QqTOF mass spectrometer (Bruker) using a CaptiveSpray source and nanoBooster at 0.20 Bar using acetonitrile. Peptides were loaded directly onto the column at a constant flow rate of 400 nl/min with buffer A (99.9% Milli-Q water, 0.1% formic acid) and eluted over 90 min using a linear gradient from 2 to 34% buffer B (99.9% acetonitrile, 0.1% formic acid). Mass spectra were acquired in a data-dependent manner, including an automatic switch between MS and MS/MS scans using a 1.5 s duty cycle and 4 Hz MS1 spectra rate followed by MS/MS scans at 8–20 Hz, dependent on precursor intensity for the remainder of the cycle. MS spectra were acquired between a mass range of 200–2000 m/z. Peptide fragmentation was performed using collision-induced dissociation. Raw files consisting of high-resolution MS/MS spectra were processed with MaxQuant (version 1.5.8.3) for feature detection and protein identification using the Andromeda search engine [22]. Extracted peak lists were searched against the *Homo sapiens* database (UniProt, October 2016), as well as a separate reverse decoy database to empirically assess the false discovery rate (FDR), using strict Trypsin specificity and allowing up to two missed cleavages. The minimum required peptide length was set to seven amino acids. In the main search, precursor mass tolerance was 0.006 Da and fragment mass tolerance was 40 ppm. The search included variable modifications of oxidation (methionine), amino-terminal acetylation, the addition of pyroglutamate (at N-termini of glutamate and glutamine) and a fixed modification of carbamidomethyl (cysteine). Peptide-spectrum matches and protein identifications were filtered using a target-decoy approach at a FDR of 1%. Protein abundance was determined according to the intensity-based absolute quantification (iBAQ) metric [23]. Gene ontology was investigated with FunRich v3.1.3 using the Gene Ontology Database [24,25]. The peptides identified by mass spectrometry were visualised using Protter [26] with membrane orientations as specified in UniProt annotations [27]. Data has been uploaded to EVpedia [28].

## Results

### Contamination and aggregation is present in EV enrichments prepared by standard differential ultracentrifugation

**Figure 2.**
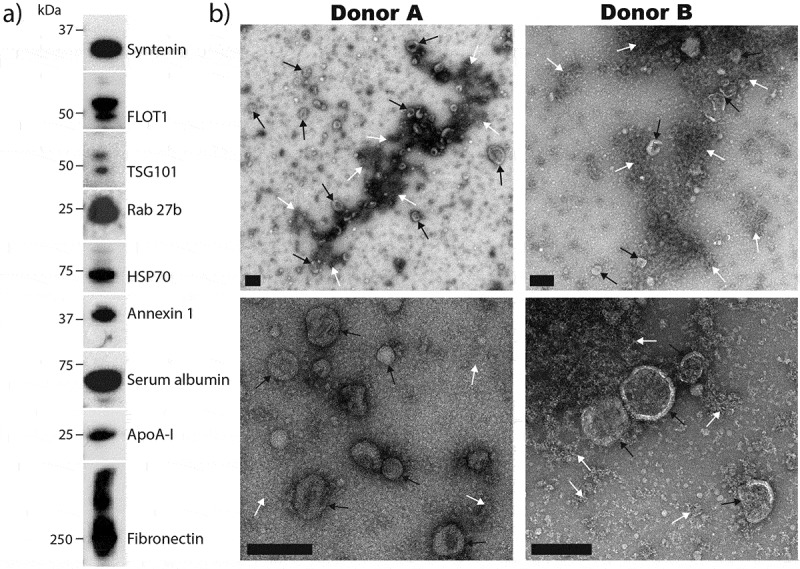
Analysis of EV enrichments from SF by differential ultracentrifugation. (a) EV pellets isolated by differential ultracentrifugation were assessed for the presence of canonical EV markers (syntenin, FLOT1, TSG101, Rab 27b, HSP70 and annexin 1) and specific contaminating proteins (serum albumin, ApoA-I and fibronectin) by western blot. Results are from a single SF donation obtained from a patient with inflammatory arthritis, and are representative of results observed with other donors. (b) Negative staining TEM analysis of differential ultracentrifugation EV isolations from two separate donors. EVs (black arrows) and amorphous material (white arrows) are indicated. Scale bars = 200 nm.

As differential ultracentrifugation is the standard means of EV preparation, we first assessed this technique for isolating EVs from SF. In western blot analysis of 100,000 x *g* ultracentrifugation pellets, EV markers (syntenin, FLOT1, TSG101, Rab 27b, HSP70 and annexin 1) were detected, confirming that EVs are present in isolations (Figure 2a). Serum albumin, the HDL marker apolipoprotein A-I (ApoA-I) and the extracellular matrix constituent fibronectin were also detected, indicating contamination with components not typically associated with EVs. Analysis of 100,000 x *g* pellets by TEM revealed structures consistent with the expected appearance of EVs (Figure 2b). However, considerable amorphous material, not associated with EVs, as well as areas of dense aggregation of EVs with amorphous material, were also observed (Figure 2b).

### Sucrose density gradient ultracentrifugation does not deplete HDLs from EV isolations

**Figure 3.**
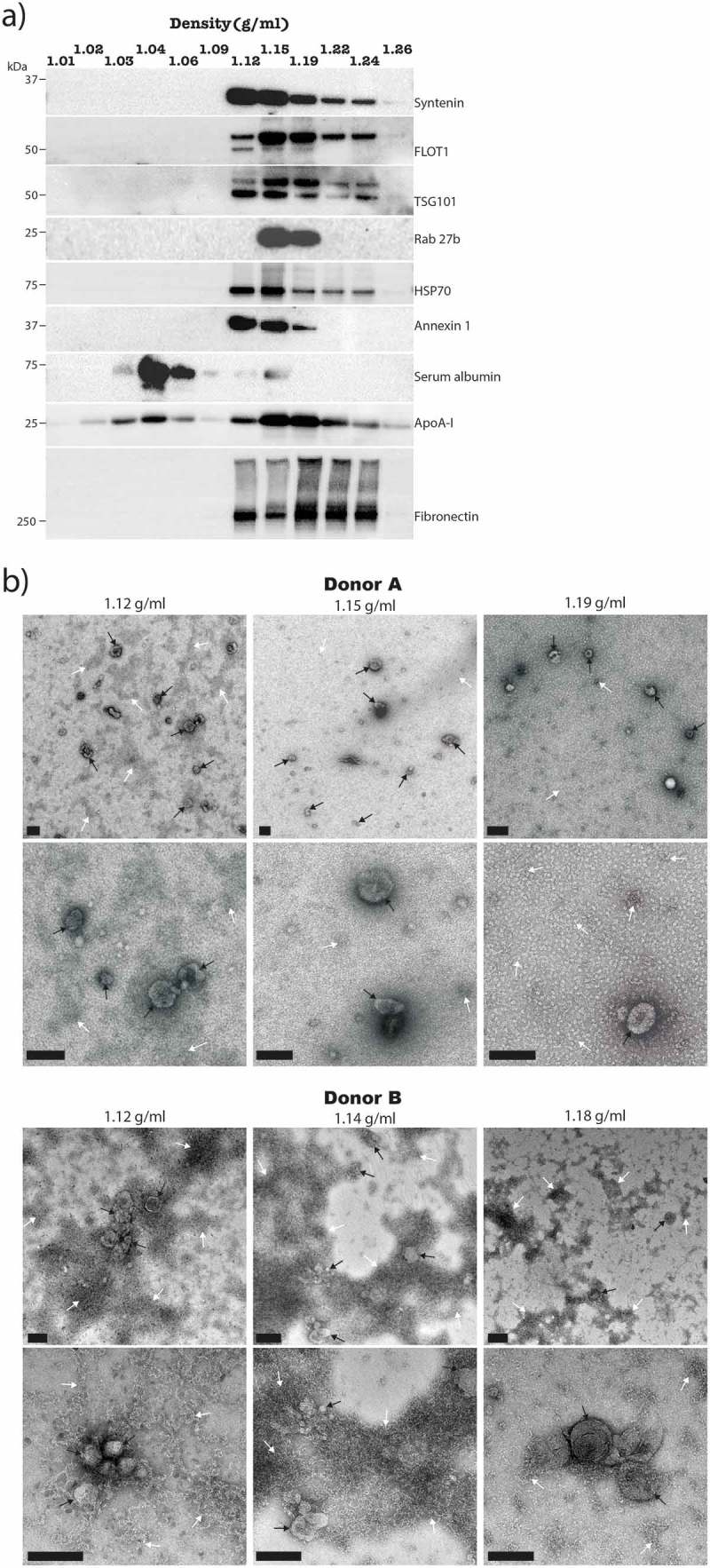
Analysis of EV enrichments from synovial fluid by sucrose density gradient ultracentrifugation. (a) Sucrose density gradient ultracentrifugation fractions were assessed for the presence of EV markers and specific contaminating proteins by Western blot with equal fraction volumes loaded. Results are from the same SF sample that was used for Figure 2b, and are representative of results observed with other donors. (b) Negative staining TEM analysis of EV containing fractions from two separate donors showing the presence of EVs (black arrows) and unspecified material (white arrows). Scale bars = 200 nm.

The efficiency of sucrose density gradient ultracentrifugation for enriching EVs from SF was assessed. When positioning the crude EV pellet, we implemented the top-down approach in an attempt to avoid potential inhibition of EV-migration through gradient medium by contaminating protein complexes [29]. In western blot analysis, EV markers were detected at sucrose densities ranging from 1.12 to 1.24 g/ml, with the greatest intensity between 1.12 and 1.19 g/ml (Figure 3a). The majority of serum albumin was detected at lower sucrose densities (1.03–1.06 g/ml), with only a small amount overlapping with EV markers. However, poor separation between ApoA-I and EV markers was still observed, confirming that density gradient ultracentrifugation is insufficient for depleting HDLs from EV isolations, as previously observed in human plasma [10]. Similarly, fibronectin also overlapped strongly with EV markers. TEM analysis of EV containing fractions revealed structures consistent with EVs as well as considerable amorphous material (Figure 3b). As with enrichments by standard differential ultracentrifugation, areas with dense aggregation of EVs with non-EV components were apparent.

### Improved EV enrichment using size exclusion chromatography

**Figure 4.**
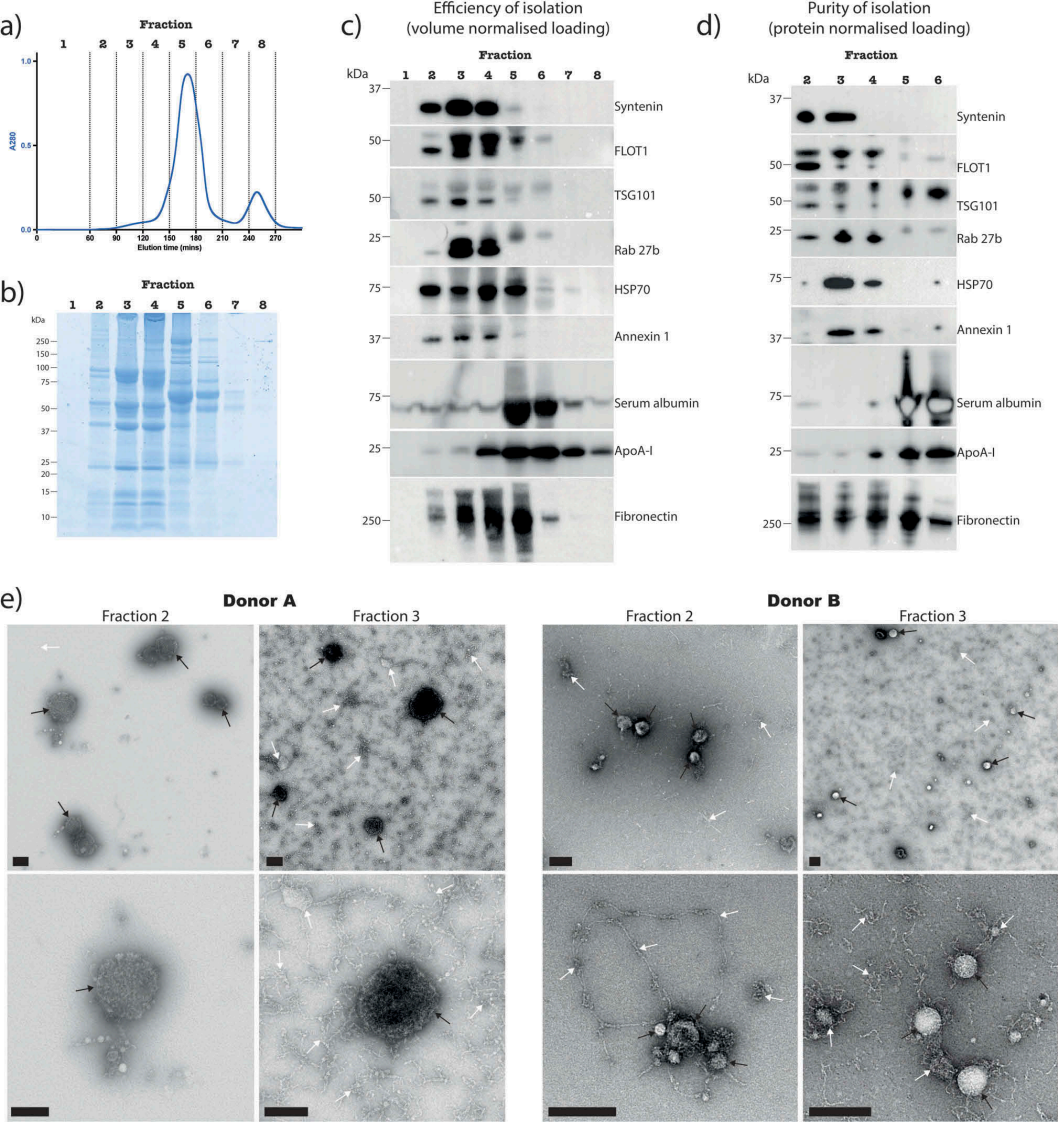
Analysis of EV enrichment from synovial fluid by SEC. (a) Chromatogram of absorbance at 280 nm versus elution time. SEC fractions were concentrated by ultracentrifugation at 100,000 x *g* and the resulting pellets assessed for: (b) protein abundance by SDS PAGE plus Coomassie staining, with loading proportional to fraction volume, (c) the presence and abundance of EV markers, serum albumin, ApoA-I and Fibronectin by Western blot, with loading proportional to fraction volume, and (d) the purity of the EV enrichment by Western blot with equal protein amount loaded into each well (fractions 1, 7 and 8 were not assessed due to insufficient amounts of protein). Results are from the same SF sample that was used for Figures 2b and 3b, and are representative of results observed with other donors. (e) Negative staining TEM analysis of non-concentrated SEC eluent from two separate donors, containing a mixture of EVs (black arrows) and contaminating material (white arrows). Scale bars = 200 nm.

A SEC-based approach for enriching EVs from SF was assessed. To achieve efficient separation of EVs from non-EV components, the HiPrep 26/60 Sephacryl S-500 HR column was selected because the column volume and resin allow for large volume sample input and small EV infiltration (respectively). The chromatogram illustrating elution time versus 280 nm absorbance profile of column eluent revealed that the majority of protein present in 10,000 x *g* SF supernatant is eluted after 150 min (Figure 4a). Eight separate fractions were collected as indicated (Figure 4a) and concentrated with 100,000 x *g* ultracentrifugation for gel electrophoresis and western blot analysis. Gel electrophoresis with Coomassie staining readily detected protein across fractions 2–7. Strikingly distinct band patterns were observed across fractions 2–4 when compared to fractions 5–7 (Figure 4b).

To assess which fractions contain the majority of EVs, the efficiency of EV isolation was assessed by western blot analysis (Figure 4c). EV markers were detected across fractions 2–4, indicating that most EVs are eluted in these fractions, separate to high amounts of protein in fractions 5–8 that co-pellet with ultracentrifugation at 100,000 x *g* (Figure 4b). The majority of serum albumin was eluted in fractions 5 and 6, whereas ApoA-I appeared in fractions 4–8. Fibronectin was detected across fractions 2–6, and was particularly abundant in fractions 3–5. The purity of EV containing fractions was then assessed by western blot (Figure 4d). EV markers were again detected across fractions 2–4, as expected. Importantly, negligible levels of serum albumin and ApoA-I appeared in fractions 2 and 3. However, fibronectin was found in all fractions analysed. A considerable proportion of EVs can therefore be collected in fractions 2 and 3, with low levels of contaminating HDLs and serum albumin. Although a portion of fibronectin eluted in EV-depleted fractions, considerable levels remained in EV containing fractions, suggesting the presence of EV-fibronectin complexes and/or contaminating extracellular matrix components containing fibronectin that are of similar size and density to EVs.

EV enriched fractions 2 and 3 were further characterised by nanoparticle tracking analysis and TEM. A size distribution profile consistent with that of small EVs (exosomes and small microvesicles) was observed (Supplementary Figure 1 and Figure 4e). TEM images of non-concentrated SEC eluent from fractions 2 and 3 confirmed the presence of EVs, with much less contaminating material than differential ultracentrifugation or sucrose density gradient ultracentrifugation preparations (Figure 4e). The contaminating material was dispersed and appeared fibrous in nature, suggesting the presence of extracellular matrix components. Importantly, as opposed to EV isolations by differential ultracentrifugation and sucrose density gradient ultracentrifugation, dense aggregation was not observed, supporting previous reports describing aggregation as a consequence of high-speed centrifugation [16].

### Comparing ultracentrifugation and ultrafiltration for concentration of the SEC eluent

Following SEC, EVs are suspended in 90 ml of SEC eluent which needs to be concentrated for downstream applications. To this end, we compared ultracentrifugation and ultrafiltration. When the eluent was concentrated by ultracentrifugation, dense aggregation re-appeared via TEM (Supplementary Figure 2) and consisted not only of clumps of amorphous extracellular material similar to those obtained via differential ultracentrifugation and sucrose density gradient ultracentrifugation (Figures 2 and 3), but also aggregation of EVs with this extracellular debris. In comparison, when SEC eluent was concentrated by ultrafiltration, extracellular debris was fibrous and dispersed and did not aggregate with EVs (Supplementary Figure 2). In summary, neither concentration technique appeared capable of depleting the non-EV fibrous-like material that remained in non-concentrated SEC eluent (Figure 4e), but ultrafiltration offered an advantage over ultracentrifugation by avoiding artefactual aggregation of EVs with these contaminants.

### Proteinase k treatment further depletes contaminating material from EV enrichments

In an attempt to further deplete contaminating material, EVs isolated from SF via SEC were treated with proteinase K or a control. An effective concentration of proteinase K was selected based on an ability to digest bovine serum albumin at a protein concentration comparable to that present in SEC EV enriched pellets (data not shown). A considerable reduction in total protein yield occurred with proteinase K treatment (Supplementary Figure 3a). Western blot analysis revealed robust depletions of fibronectin following proteinase K treatment (Supplementary Figure 3b) and, consistent with this, TEM analysis confirmed a depletion of contaminating material (Supplementary Figure 3c). Proteinase K treatment also resulted in lower levels of all EV markers that were analysed by volume normalised western blot, including the EV luminal proteins TSG101 and annexin 1 (Supplementary Figure 3b), suggesting possible loss of EVs as well.

### Mass spectrometry analysis of EV enriched proteins

**Figure 5.**
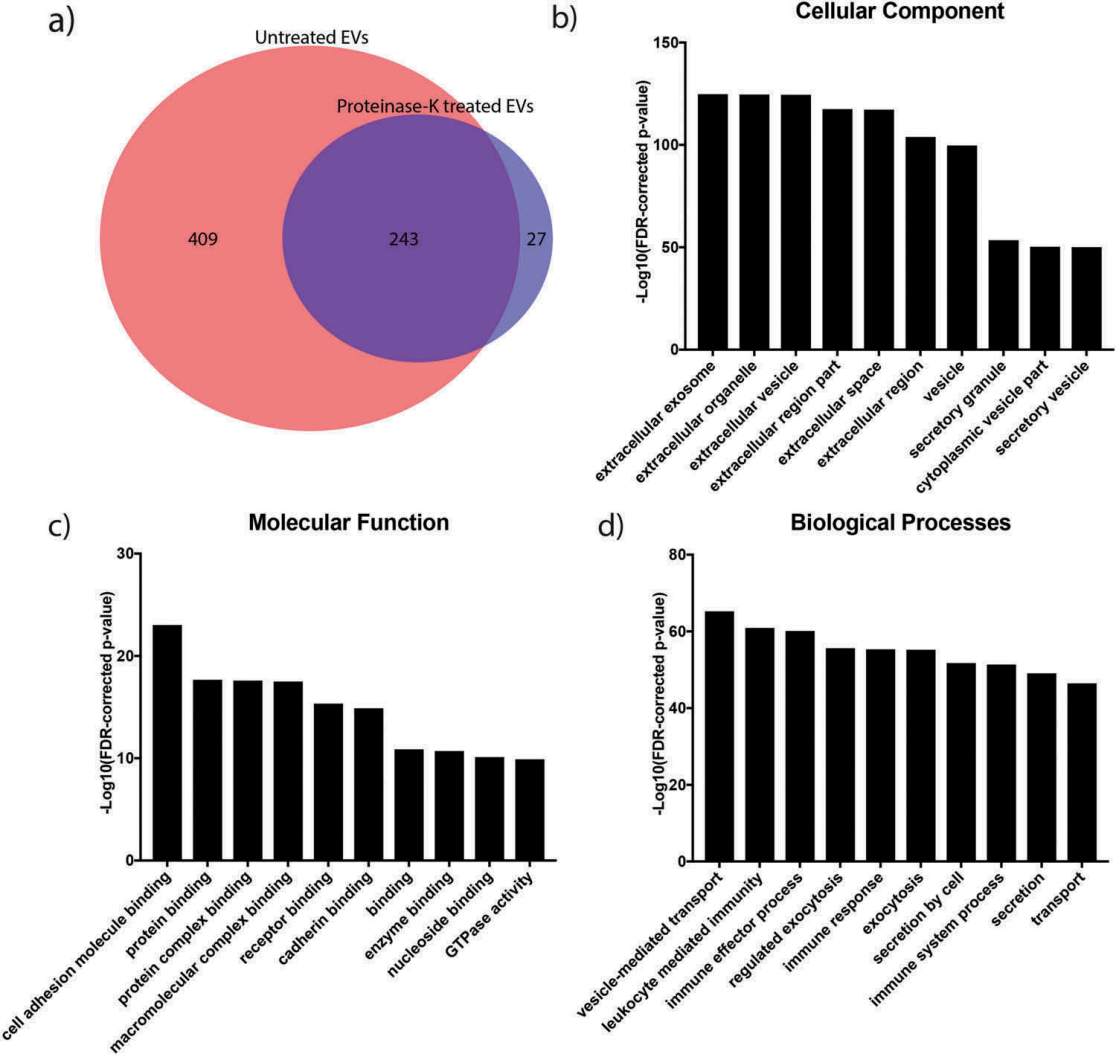
Proteomic analysis of EVs isolated from synovial fluid of a rheumatoid arthritis patient. (a) Venn diagram comparing proteins identified by mass spectrometry analysis of SEC EVs in the presence or absence of proteinase K. About 652 unique proteins were identified without proteinase K treatment, and this number reduced to 270 following the addition of proteinase K. (b–d) GO analysis of the proteins identified from mass spectrometry analysis of proteinase K treated sample including cellular function and biological processes.

To accurately characterise the protein content of SF EVs, EVs isolated from RA SF via SEC were treated with proteinase K and then subjected to high-resolution mass spectrometry analysis. 270 proteins were identified by mass spectrometry in the sample treated with proteinase K (Figure 5a), which was considerably less than the 652 proteins identified in the untreated control. Across both samples, a total of 679 unique proteins were identified, of which 243 were present in both the proteinase K treated and untreated samples.

The 270 proteins remaining in the proteinase K treated isolation are likely to contain EV enriched proteins, as the EV membrane should protect the cargo from proteinase K-mediated degradation. Indeed, in the 270 proteins found in the proteinase K treated sample there was robust enrichment for proteins annotated as EV-associated (Supplementary Table 2 and Figure 5b). Moreover, comparison of the cellular origin of proteins between the proteinase K treated and untreated samples showed >10-fold enrichment for proteins derived from the endosomal membrane (consistent with being exosomal) as well as an almost 10-fold depletion of extracellular matrix proteins (Supplementary Figure 4). Further gene ontology analyses of the 270 proteins revealed an over-representation of immunologically-related biological processes as well as proteins involved in cellular adhesion and protein binding (Figure 5c-d). Importantly, 27 proteins were uniquely identified in the proteinase K-treated isolation, indicating that proteinase K treatment improves detection of EV enriched proteins that are otherwise masked by contaminating proteins.

The 652 proteins identified in the untreated sample are likely to include not only EV proteins but also contaminants. Consistent with this, the 20 most abundant proteins identified in the untreated EV sample consisted almost entirely of extracellular matrix and immunoglobulin associated components (Table 1). In contrast, the 20 most abundant proteins detected in the proteinase K-treated sample included canonical EV proteins, CD63 and MHC components, confirming that proteinase K treatment improves identification of proteins enriched in EVs (Table 2). Interestingly, the vast majority of proteins identified in the untreated sample have been previously identified in datasets reported by EVpedia (Supplementary Table 3), suggesting that many of these datasets are also likely to contain contaminating proteins.

**Table 1.** About 20 most abundant proteins in an untreated SEC EV enrichment from human RA SF as analysed by mass spectrometry.

Protein names	Gene names	iBAQ score
Fibrinogen gamma chain	FGG	5.6E+07
Fibrinogen beta chain	FGB	4.6E+07
Ig gamma-1 chain C region	IGHG1	2.3E+07
Ig kappa chain C region	IGKC	2.3E+07
Ig lambda-3 chain C regions	IGLC3	2.2E+07
Fibrinogen alpha chain	FGA	1.5E+07
Histone H2A type 1-J	HIST1H2AJ	1.5E+07
Ig kappa chain V-III region SIE	IGKV3-20	1.5E+07
Ig mu chain C region	IGHM	1.3E+07
Histone H4	HIST1H4A	1.3E+07
Fibronectin	FN1	1.3E+07
Clusterin	CLU	8.4E+06
Immunoglobulin heavy variable 1-18	IGHV1-18	8.1E+06
Immunoglobulin lambda variable 3-19	IGLV3-19	7.7E+06
Galectin-3-binding protein	LGALS3BP	7.2E+06
Ig kappa chain V-III region CLL	IGKV3-15	6.8E+06
Vitronectin	VTN	6.8E+06
Ig alpha-1 chain C region	IGHA1	5.8E+06
Ig kappa chain V-IV region	IGKV4-1	5.4E+06
Ig heavy chain V-II region ARH-77	IGHV4-61	4.5E+06

**Table 2.** 20 most abundant proteins in a proteinase K-treated SEC EV enrichment from human RA SF as analysed by mass spectrometry.

Protein names	Gene names	iBAQ score
Tetraspanin;CD63 antigen	CD63	2.5E+06
Tyrosine-protein kinase Fer	FER	1.8E+06
Ubiquitin-60S ribosomal protein L40	UBB	1.8E+06
Oxysterol-binding protein-related protein 2	OSBPL2	1.8E+06
Fibrinogen beta chain	FGB	1.2E+06
Ig mu chain C region	IGHM	6.8E+05
HLA class II histocompatibility antigen, DRB1-4 beta chain	HLA-DRB1	5.1E+05
Complement component C9	C9	5.0E+05
HLA class II histocompatibility antigen, DR alpha chain	HLA-DRA	4.8E+05
Immunoglobulin lambda variable 3-25	IGLV3-25	4.1E+05
Ferritin light chain	FTL	3.6E+05
Proteolipid protein 2	PLP2	3.5E+05
Peptidyl-prolyl cis-trans isomerase A	PPIA	3.1E+05
BH3-interacting domain death agonist	BID	3.1E+05
Protein S100-A9	S100A9	3.0E+05
Complement component C8 alpha chain	C8A	3.0E+05
Rho GDP-dissociation inhibitor 2	ARHGDIB	2.6E+05
Ras-related protein Rap-1b	RAP1B	2.3E+05
Actin, cytoplasmic 1	ACTB	2.0E+05
HLA class I histocompatibility antigen, B-18 alpha chain	HLA-B	1.9E+05

In comparing the proteins identified in the proteinase K-treated and untreated samples, it was also evident that proteinase K resulted in loss of some canonical EV proteins (e.g. CD9 and CD81), consistent with our earlier western blotting results (Supplementary Figure 3b). We hypothesised that this selective loss might be due to the fact that mass spectrometry only detects some peptides within a protein and that, for some membrane-bound proteins, the presence of these peptides on the exposed external surface of the EV membrane might render them “invisible” following proteinase K treatment. To test this, we mapped the locations of peptides detected by mass spectrometry to their location on a range of classic EV proteins. First, we assessed peptides for annexin 1, which mainly localises to the inner leaflet of the EV membrane. These mapped to the protected, intraluminal surface and, as expected, were still detectable following proteinase K treatment (Supplementary Figure 5). Next, we assessed peptides for the tetraspanins, CD9 and CD81, both of which were only identified by mass spectrometry in the absence of proteinase K. Consistent with our hypothesis, in the absence of proteinase K, detected peptides for CD9 and CD81 were exclusively localised to domains annotated as external to the outer EV membrane [27], and proteinase K treatment resulted in loss of these peptides and failure to detect either of these proteins (Supplementary Figure 5). Finally, we examined CD63, which was enriched following proteinase K treatment. Unexpectedly, peptides for CD63 mapped to domains annotated as external to the outer EV membrane in the presence and absence of proteinase K (Supplementary Figure 5), suggesting that this region is protected from proteinase K degradation (e.g. on account of its extensive glycosylation [30,31]), or that CD63 might exist with an “inside-out” topology as has been described for other EV membrane proteins [32].

## Discussion

Here we have shown that SEC-based EV enrichments from human SF are of greater purity than can be achieved by differential ultracentrifugation and sucrose density gradient ultracentrifugation. Specifically, the SEC protocol presented here is capable of depleting serum albumin and HDLs from EV enrichments, which otherwise remain in ultracentrifugation-based methods. We also show that a considerable proportion of the protein that pellets with ultracentrifugation is not associated with EVs and can be separated from EV enrichments using SEC. Finally, we have demonstrated that proteinase K treatment of SEC EV isolations reduces extracellular proteins, with proteomic profiling of proteinase K treated EV pellets revealing EV incorporated proteins.

The presence of contaminating material and artefactual aggregation observed in EVs isolated using ultracentrifugation-based methods highlights the limitations of these techniques for the study of EVs. Moreover, these limitations raise questions about previously published observations, since the identification of EV content, interactions and functional effects is obviously confounded by isolation methods.

For example, HDLs have been shown here, and by others, to contaminate EVs prepared using ultracentrifugation-based methods [10]. Like EVs, HDLs are carriers of proteins [33] and RNAs [34], and are capable of functionally interacting with recipient cells [35,36]. Insufficient depletion of HDLs from EV preparations might therefore result in the content and function of HDLs being mistakenly attributed to EVs. Similarly, EVs isolated by high-speed centrifugation from the SF of patients with rheumatoid and osteoarthritis have previously been described as containing a wide range of circulating proteins [6], and to induce the release of pro-inflammatory cytokines in recipient synovial fibroblasts [37]. Given limitations in the method of EV isolation and lack of corroborative evidence, observations attributed to EVs in these studies (and others) may be spurious. Another potentially functional contaminant of EV preparations is fibronectin. Although well known as an extracellular matrix protein, fibronectin has been described to interact with EVs via EV-associated integrins [38], and so it is perhaps not surprising that fibronectin was detected in our EV isolations. However, considerable amounts of fibronectin were also present in ultracentrifugation pellets of *EV-deficient* SEC fractions, suggesting that the large amount of non-EV-associated fibronectin that exist in SF cannot be separated from EV-associated fibronectin by ultracentrifugation-based methods. Given that pro-inflammatory activities for EV-fibronectin complexes have been reported [39], artefactual co-isolation of fibronectin and EVs may have confounded past experimental observations [37].

Even with SEC, contamination of EVs with extracellular proteins remained an issue, albeit improved by proteinase K. Notably, almost all of the contaminating, proteinase K-sensitive extracellular proteins that we observed after SEC-based EV enrichment have been previously identified in multiple datasets uploaded to EVpedia. This suggests that contamination is a widespread problem and emphasises the need for comprehensive characterisation of EV isolations and careful experimental design, so that experimental observations can be reliably attributed to EVs [40].

Our proteomic analyses of EVs isolated from SEC provide insights into the content and function of RA SF EVs. For example, the presence of Tyrosine-protein kinase Fer as one of the most abundant EV proteins and second only to CD63 (Table 2) suggests kinase activity within SF EVs, while the presence of Ubiquitin-60S ribosomal protein L40 supports reported roles for ubiquitin in protein trafficking to exosomes [41–43]. Unexpectedly, the Membrane Attack Complex components, Complement C9 and C8α, were also identified, suggesting EVs may be involved in facilitating complement-mediated immune responses in RA SF. Interestingly, fibrinogen β-chain remained abundant in the proteinase K-treated sample, supporting a proposed role for EV-associated citrullinated fibrinogen in propagating autoimmune responses in RA SF [1,6]. More studies involving larger cohorts of patients will be required to further investigate if these observations are important in RA pathogenesis.

There are certain limitations to our study. Notably, a number of residual proteins not expected to be incorporated into EVs, including ApoA-I and fibronectin, still remained in the EV isolation following proteinase K treatment, indicating that proteinase K was unable to access all cleavage sites, and that some contaminating material, including HDLs, persist at lower levels. Thus, while enrichment for canonical EV components (e.g. CD63) following proteinase K treatment provides confidence that our method is capable of identifying EV proteins, some proteins may still not be truly EV incorporated. Another concern is that the processing of SF may activate platelets [44], which could artefactually increase SF fibrinogen, fibronectin and immunoglobulins [45], as well as platelet-derived EVs [46].

Proteinase K treatment may also impair EV integrity. In support of this, we observed that proteinase K decreased the abundance of EV luminal proteins, which one might expect would be protected from proteinase K degradation. We hypothesise that this is due to loss of EVs, possibly due to cleavage of EV membrane proteins and a subsequent reduction in membrane integrity, as can occur during proteinase K treatment of whole cells [47]. Considering that proteinase K treatment also affects the bioactivity of EVs [48], experiments on EV function and/or surface protein composition would benefit from other methods of contaminant depletion. For example, an additional wash and ultracentrifugation step following SEC, or a combination of density gradient ultracentrifugation with SEC would avoid proteinase K associated damage. In addition, other approaches not tested here – including use of a bottom-up density gradient and/or iodixanol as the density gradient medium [49] – might also prove valuable for depleting contaminants from SF EV isolations.

## Conclusion

The involvement of SF EVs in joint physiology and pathophysiology – particularly in inflammatory arthritis – is increasingly recognised [5,50–52]. However, difficulties in obtaining high quality EV enrichments from SF complicates investigation into the content and function of SF EVs. The SEC technique presented here is an efficient method for EV enrichment that results in greater purity than traditional ultracentrifugation-based approaches. Our technique will allow improved identification of EV-associated molecules that may be dysregulated or modified in the SF of patients with joint diseases such as RA, some of which could represent novel diagnostic or therapeutic targets, and is likely to be useful for EV isolation from other complex biological fluids.
